# Subconjunctival Loiasis

**DOI:** 10.4269/ajtmh.2011.10-0526

**Published:** 2011-02-04

**Authors:** Alejandro Lichtinger, Mauricio Caraza, Michael Halpert

**Affiliations:** Hadassah-Hebrew University of Jerusalem and La Paz Hospital, Equatorial Guinea

A 23-year-old man presented to La Paz Hospital complaining of a “worm moving in his eye.” He was otherwise healthy. On examination under natural light a mobile worm was seen in the superior subconjunctival space. When exposed to the bright light/heat of the slit lamp it migrated to the fornix; moments later the worm was visible again and every time it was exposed to the light it would disappear. A live, 2 cm in length worm was removed through a small conjunctival incision.

In a second case an 18-year-old woman presented complaining of foreign body sensation. We found a dead worm 4.7 cm in length, which was excised from the inferior subconjunctival space, where it was surrounded by fibrotic tissue.

Loiasis is endemic in this area of Africa. Humans get the *Loa loa* larvae from the bite of infected chrysops flies; the larvae mature into adult worms that travel around the patient's subcutaneous tissue and occasionally the subconjunctival space causing localized inflammation. The worm can move at speeds of up to 1 cm per minute as seen in our first case. The worm roamed to the fornix every time it was exposed to the light/heat of the slit lamp.

**Figure 1.**
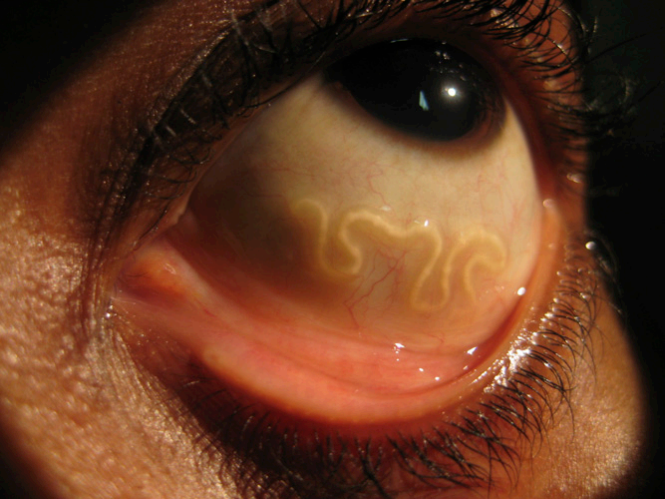
Dead subconjunctival *Loa loa* worm from the second case.

## Supplementary Material

Supplementary Figure

[Supplemental figures]

