# Supplementary material for: Subconjunctival Loiasis
Source: Am J Trop Med Hyg. 2011 Feb 4;84(2):183. doi: 10.4269/ajtmh.2011.10-0526 (PMC3029165; doi:10.4269/ajtmh.2011.10-0526)
Supplement: Supplementary Figure [file SD4.pdf]

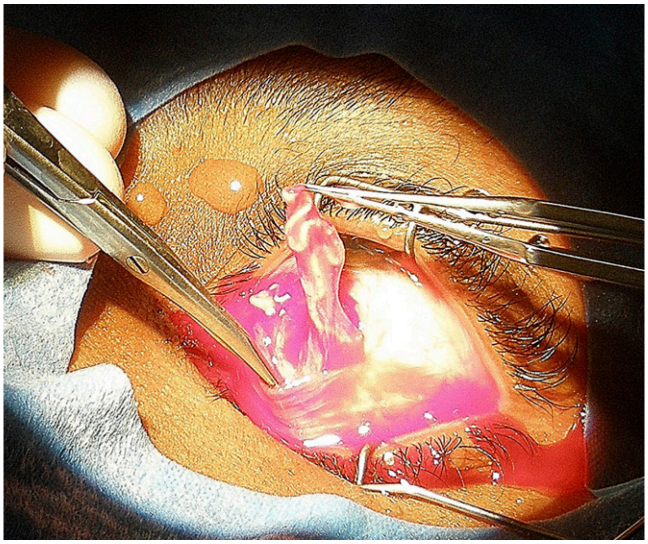

Figure S1. Surgical excision of dead subconjunctival worm surrounded by fibrotic tissue.

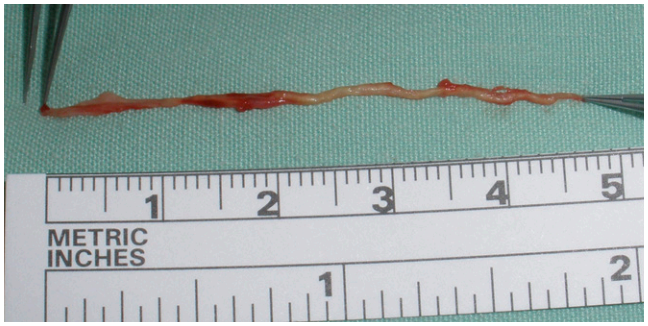

Figure S2. Excised worm 4.7 cm in length.

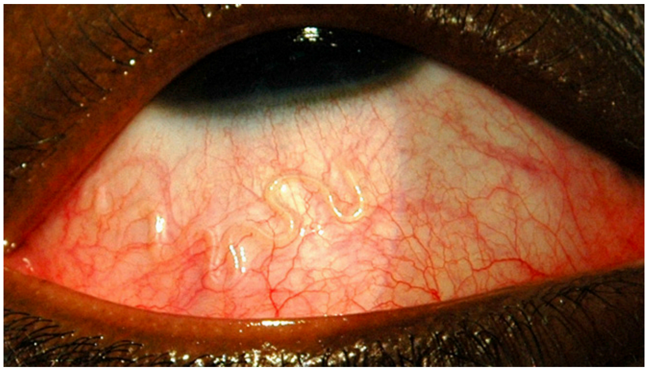

Figure S3. Live subconjunctival mobile worm from the first case.

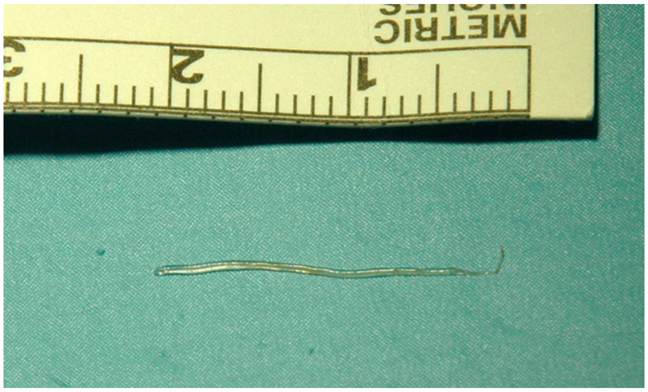

Figure S4. Excised worm 2 cm in length.
