# Supplementary material for: Subconjunctival Loiasis
Source: Am J Trop Med Hyg. 2011 Feb 4;84(2):183. doi: 10.4269/ajtmh.2011.10-0526 (PMC3029165; doi:10.4269/ajtmh.2011.10-0526)
Supplement: [Supplemental figures] [file supp_84_2_183__index.html]

 Subconjunctival Loiasis -- Lichtinger et al. 84 (2): 183 Data Supplement - Supplemental figures -- American Journal of Tropical Medicine and Hygiene **Subconjunctival Loiasis**  
 Am J Trop Med Hyg Lichtinger et al. 84: 183

## Supplemental figures

**Files in this Data Supplement:**

- Supplemental figures
